# Chronic Rhinosinusitis with Polyps Is Characterized by Increased Mucosal and Blood Th17 Effector Cytokine Producing Cells

**DOI:** 10.3389/fphys.2017.00898

**Published:** 2017-12-19

**Authors:** Dijana Miljkovic, Alkis J. Psaltis, Peter J. Wormald, Sarah Vreugde

**Affiliations:** Department of Surgery-Otolaryngology Head and Neck Surgery, University of Adelaide, Adelaide, SA, Australia

**Keywords:** chronic rhinosinusitis, flow cytometry, nasal polyps, Th17, Th17 cytokines

## Abstract

**Background:** Recent studies have implied a role for Th17 cells in CRS with nasal polyps (CRSwNP) patients. However, the capacity of these cells to produce Th17 cytokines is still unknown. Here we sought to quantify IL-17A, IL-17F, IL-21, and IL-22 cytokines produced by Th17 cells in mucosal tissue and peripheral blood of CRSwNP, CRS without nasal polyps (CRSsNP) and control patients.

**Methods:** Samples were prospectively collected from CRS patients and non-CRS controls. We used flow cytometry to characterize the Th17 cells and their cytokines in sinonasal tissue and peripheral blood.

**Results:** A total of 36 patients were recruited to the study. CRSwNP patients had significantly more tissue IL-17A (9.53 ± 2.71 vs. 1.11 ± 0.43 vs. 0.77 ± 0.07), IL-17F (4.96 ± 1.48 vs. 0.88 ± 0.31 vs. 0.56 ± 0.04), IL-21 (5.55 ± 2.01 vs. 1.60 ± 0.71 vs. 1.53 ± 0.55) and IL-22 (4.73 ± 1.58 vs. 0.70 ± 0.28 vs. 0.88 ± 0.26) producing Th17 cells compared to CRSsNP and control mucosa per mg of tissue, respectively. Allergic CRSwNP patients had decreased numbers of IL-21 producing Th17 cells compared to non-Allergic CRSwNP. (1.69 ± 0.57 vs. 9.41 ± 3.23) per mg of tissue, respectively (Kruskal-Wallis *p* < 0.05).

**Conclusion:** In summary our study identified increased numbers of IL-17A, IL-17F, IL-21 and IL-22 positive Th17 cells in CRSwNP patient polyps and peripheral blood suggesting an altered activation state of those cells both locally and systemically. Atopic CRSwNP had decreased amounts of tissue Th17 cell derived IL-21 implying a potential protective role for IL-21 in CRSwNP allergic inflammation.

## Introduction

T helper 17 (Th17) cells are characterized by CC motif 6 chemokine receptor (CCR6) expression and produce the signature cytokines interleukin-17A (IL-17A), IL-17F, IL-21, and IL-22. Th17 cells play a significant role in the adaptive immune system by generating inflammation in response to infection, in particular to *Candida albicans* and *Staphylococcus aureus* (Brucklacher-Waldert et al., 2009). Both IL-17A and IL-17F cause the up-regulation of pro-inflammatory cytokines and chemokines such as IL-6, granulocyte colony-stimulating factor (GCSF), and CXCL1 and CXCL2 (Fossiez et al., 1996). In the airways, IL-17A has been shown to cause a release of chemokines that recruit neutrophils and fungicidal peptides (Laan et al., 1999; Kinugasa et al., 2000; O'Connor et al., 2009; Puel et al., 2012; Lee et al., 2015). Although vital in protecting the host against pathogens, dysregulated inflammation, if sustained, may result in inflammation-associated pathologies such as tissue damage and the disruption of mucosal homeostasis. Excessive IL-17A production in the synovial fluid of rheumatoid arthritis patients has been demonstrated to have a role in the progression of the disease (Siloşi et al., 2016) Antibodies targeting IL-17A result in a reduction of clinical symptom severity, further highlighting the importance of aberrant cytokine responses in differing immune microenvironments (Hueber et al., 2010).

IL-21 intensifies the production of pro-inflammatory cytokines in the mucosa as well as aiding in the recruitment of neutrophils (De Nitto et al., 2010; Neurath, 2014). In the gut mucosa it is considered to be pathogenic and antibodies targeted to neutralizing the cytokine have a protective and anti-inflammatory effect (Yu et al., 2015). IL-21 is also implicated in a range of autoimmune diseases. It has been shown to be increased in active systemic lupus erythematosus (SLE) and could be responsible for the generation of plasma cells in the disease state (Nakou et al., 2013) and epithelial cell activation and survival by moderating T regulatory cell subsets (Lin et al., 2014). IL-22 is also capable of inducing proliferative and anti-apoptotic pathways as well as producing antimicrobial peptides which help prevent tissue destruction and promote repair (Kim et al., 2014).

Th17 responses have been implicated in the pathophysiology of a number of inflammatory disorders, including rheumatoid arthritis, SLE, and asthma. In chronic rhinosinusitis (CRS), studies have assessed Th17 abundance by measuring IL-17 expression. Although early studies did not find an increase in IL-17 production in CRS (Van Bruaene et al., 2008; Cao et al., 2009) two more recent studies in the adult Chinese population have reported increased IL-17 in eosinophilic CRSwNP, suggesting a possible role of Th17 cells in this inflammatory condition (Cao et al., 2009; Wei et al., 2014). However, it has been shown that IL-17 can also be produced by different immune cell types, including neutrophils and gammadelta T cells and thus, differential IL-17 abundance does not necessarily imply changes in Th17 cell frequencies (Coffelt et al., 2015). Recently, we have reported an increase in Th17 cell numbers in CRS with polyp (CRSwNP) patients (Miljkovic et al., 2016). In this present study we further characterize the local and peripheral Th17 cell cytokine response in CRSwNP, CRS without nasal polyps (CRSsNP) and controls.

## Methods

### Patient sample collection

This study was approved by the Human Research Ethics Committee of the Queen Elizabeth Hospital, Adelaide, Australia and all patients provided written informed consent prior to enrollment. Specimens were prospectively collected at the time of endoscopic sinus surgery from non-diseased controls and consecutive CRS patients (CRSsNP and CRSwNP). Polyp tissue was collected from CRSwNP patients with ethmoid mucosa obtained from CRSsNP and controls. All patients also had peripheral blood collected prior to their operation. Control patients were undergoing endoscopic skull base procedures procedures and had no clinical, endoscopic or radiologic evidence of past or present sinonasal disease. CRS patients fulfilled the diagnostic criteria of the position papers of American Academy of Otolaryngology and Head and Neck Surgery and the European Position Statement on Chronic Rhinosinusitis (Fokkens et al., 2012a; Lin and Nnacheta, 2015). Patients with CRS were further sub-classified according to the absence or presence of polyps on nasal endoscopy. Exclusion criteria included minors < 18 years of age, pregnancy, malignancy, immune disorders, and the use of antihistamines, antibiotics, immune-modulating drugs or oral corticosteroids in the month preceding surgery. Patients were classified as atopic if they had positive RAST and/or skin prick testing. Demographic and clinical data was collected from all patients prior to the commencement of the study.

### Cell preparation

Tissue samples from the ethmoid mucosa were washed and dissected into pieces ≤ 2 mm before being prepared into a single cell suspension by enzymatic digestion with 2 mg/ml collagenase type II (Sigma-Aldrich, MO, USA) and 0.04 mg/ml DNAse I (Roche Applied Sciences, Vilvoorde, Belgium) for 45 min at 37°C. Cell suspensions were filtered through a 70 μm nylon mesh and washed in PBS. Lymphocytes were isolated from heparinized peripheral blood by Ficoll-Paque PLUS (GE Healthcare, Chicago, USA) (Miljkovic et al., 2014).

### Flow cytometric immunophenotyping

Cells stimulated with cell stimulation cocktail (eBioscience, San Diego, CA, USA) for 6 h were washed and stained with Fixable Viability Dye eFluor® 506 (eBioscience, San Diego, CA, USA) at 4°C for 30 min to exclude dead cells before cell surface staining with CD4+CCR6+CD45+ to define Th17 cells (Pandya et al., 2016). Cells were fixed and permeabilised before staining with intracellular cytokines IL-17A, IL-17F, IL-21, and IL-22 (eBioscience, San Diego, CA, USA). Antibody details are listed in Supplementary Table S1. Gates were set on unstimulated and fluorescent minus one controls shown in Supplementary Figure S1.

### Statistical analysis

The data were summarized using means with standard deviations and medians with range. The Kruskal-Wallis test was used for the comparison of data from the three independent disease groups of patients. Mann-Whitney test was used for comparison of two independent disease groups. All tests were two-tailed and significance was assessed at the 5% alpha using GraphPad Prism 7.

## Results

### Patients

Samples from a total 36 patients (12 CRSwNP, 19 CRSsNP and 5 controls) were used for this study. Patient demographic information is summarized in Supplementary Table S2.

### Th17 cells are increased in CRSwNP mucosa

**Figure 1:**
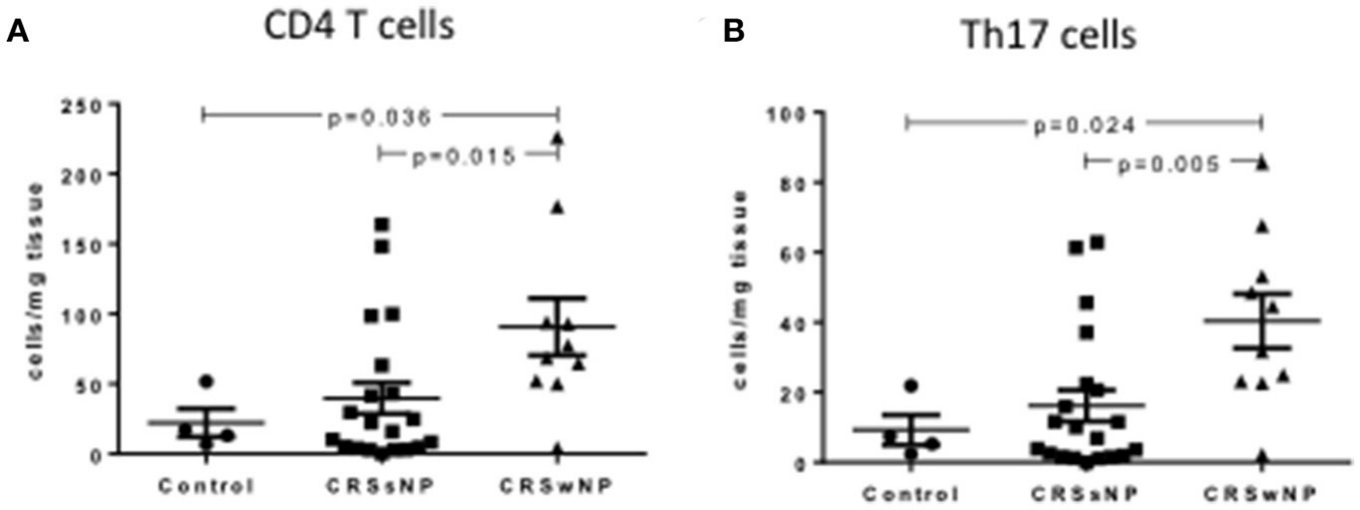
Percentage of CD4+ **(A)** and Th17 cells **(B)** per mg of tissue in control, CRSsNP and CRSwNP mucosa. Medians with interquartile range. *P* ≤ 0.05 Kruskal-Wallis.

CRSwNP patients had significantly more total tissue CD4+ T helper cells compared to CRSsNP and control mucosa (90.73 ± 20.47 vs. 39.72 ± 11.14 vs. 22.24 ± 10.07 per mg of tissue respectively, Kruskal-Wallis *p* < 0.01) (Figure 1A). CRSwNP patients had more Th17 cells in the polyps compared to CRSsNP and control mucosa (40.39 ± 7.75 vs. 16.19 ± 4.49 vs. 9.26 ± 4.32 per mg of tissue respectively, Kruskal-Wallis *p* < 0.01) (Figure 1B). No differences were seen in CD4+ or Th17+ cell numbers as a % of CD45+ cells in the peripheral blood of control, CRSsNP or CRSwNP patients.

### Th17 cytokines are increased in mucosal CRSwNP Th17 cells

**Figure 2:**
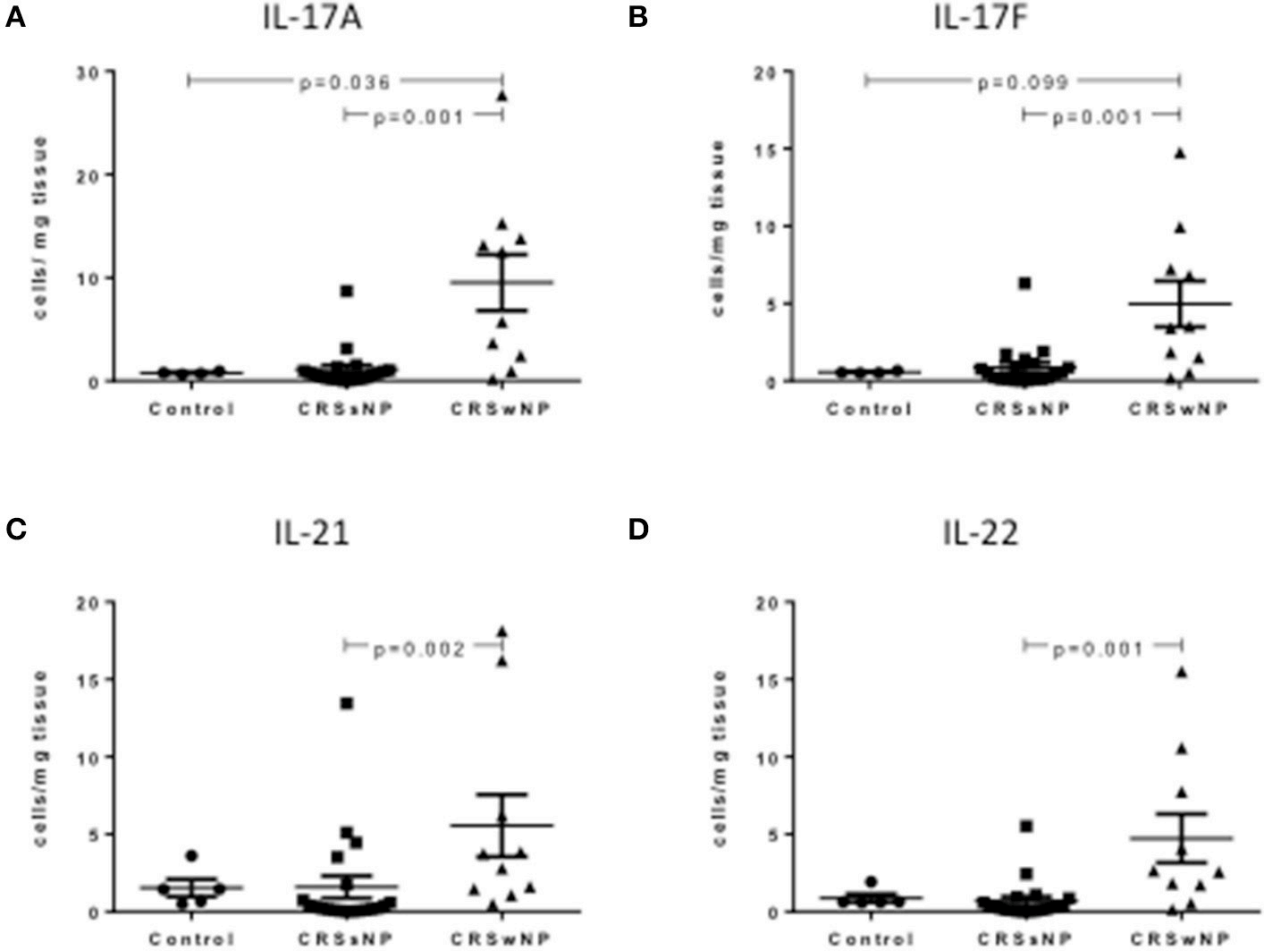
Percentage of IL-17A+Th17+ **(A)**, IL-17F+Th17 **(B)**, IL-21+Th17 **(C)**, and IL-122+Th17 **(D)** cells per mg of tissue in control, CRSsNP and CRSwNP mucosa. Medians with interquartile range. *P* ≤ 0.05 Kruskal-Wallis.

Intracellular staining of Th17 cells showed a significant increase in Th17 cytokine producing Th17 cells per mg of tissue in CRSwNP patients compared to CRSsNP and/or control mucosa, including IL-17A, IL-17F, IL-21, and IL-22 (Kruskal-Wallis *p* < 0.01, Figures 2A–D). Supplementary Table S3 details cytokine expressing Th17 cells for controls, CRSsNP and CRSwNP patients.

### Th17 cytokines are increased in peripheral CRSwNP Th17 cells

**Figure 3:**
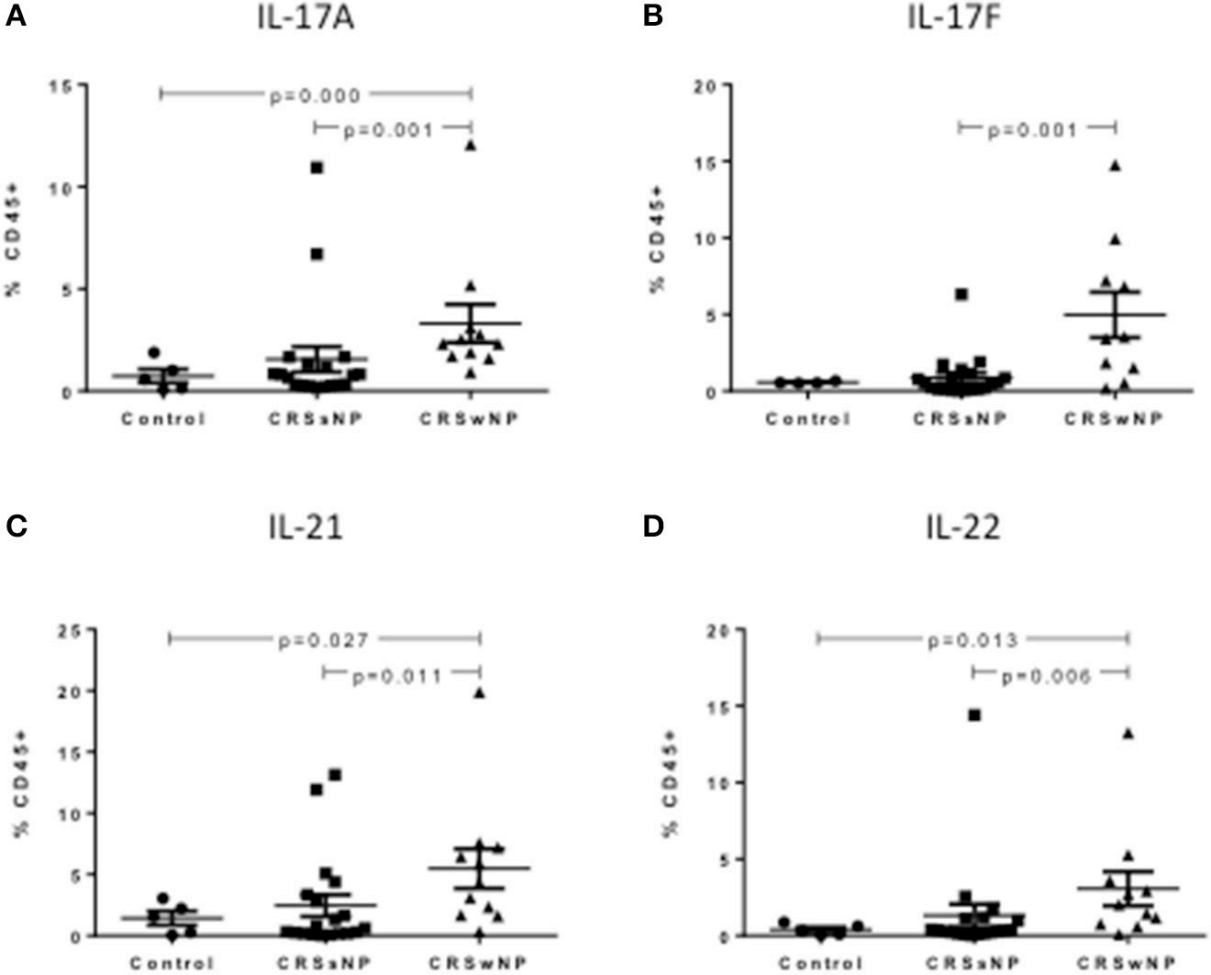
Percentage of IL-17A+Th17 **(A)**, IL-17F+Th17 **(B)**, IL-21+Th17 **(C)**, and IL-22+Th17 **(D)** cells as a % of CD45+ cells in control, CRSsNP and CRSwNP peripheral blood. Medians with interquartile range. *P* ≤ 0.05 Kruskal-Wallis.

In the peripheral blood, CRSwNP patients had significantly more Th17 cells producing IL-17A, IL-17F, IL-21, and IL-22 compared to CRSsNP and control patients (Kruskal-Wallis *p* < 0.01, Figures 3A–D). Supplementary Table S4 details cytokine expressing Th17 cells for controls, CRSsNP and CRSwNP patients.

### Th17 cytokines were equally produced within control, CRSsNP and CRSwNP mucosa, and peripheral blood

**Figure 4:**
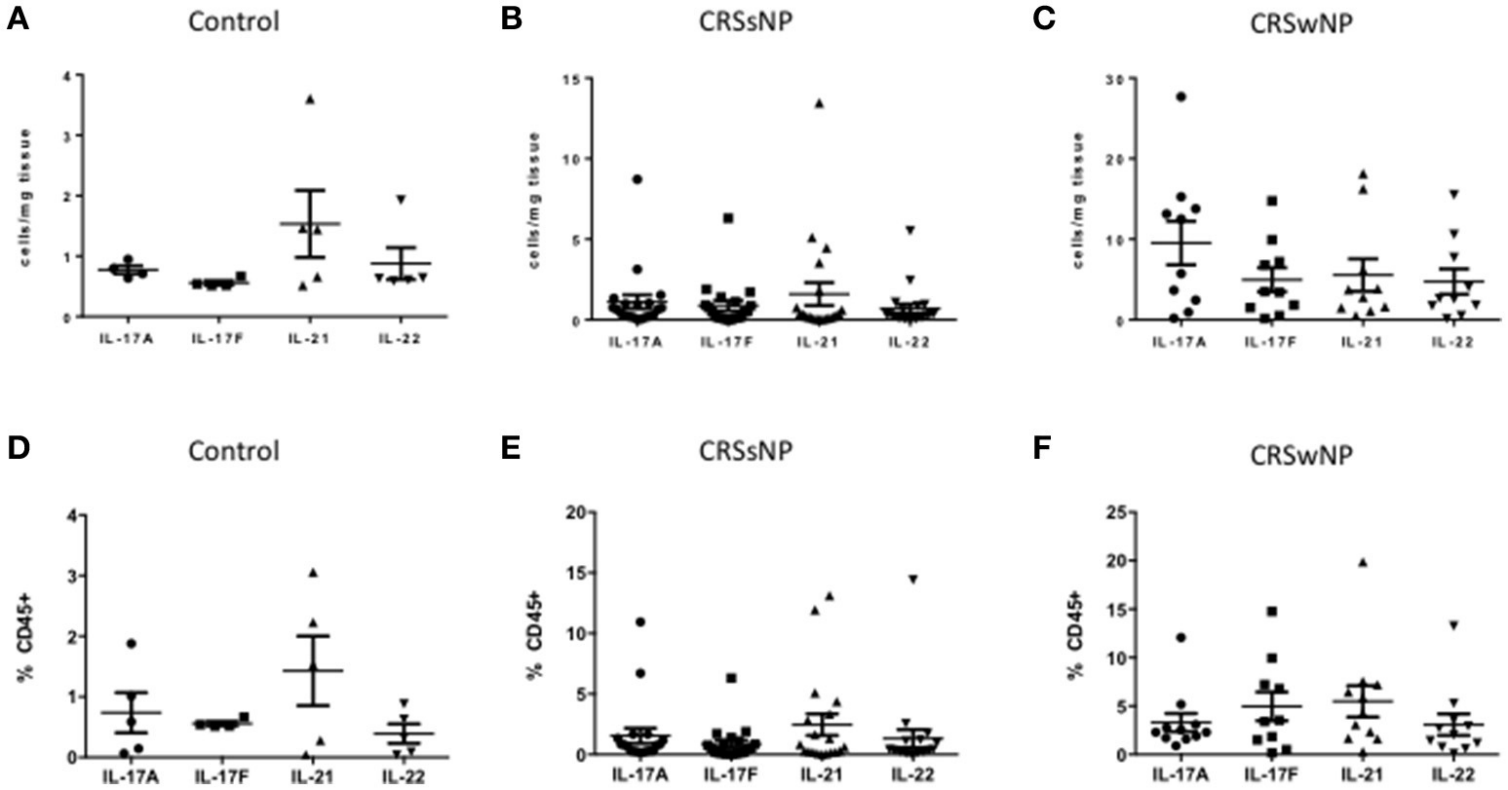
Percentage of IL-17A, IL-17F, IL-21, and IL-22 Th17 cells in control **(A)**, CRSsNP **(B)** and CRSwNP **(C)** mucosa and control **(D)**, CRSsNP **(E)** and CRSwNP **(F)** peripheral blood. Medians with interquartile range. *P* ≤ 0.05 Kruskal-Wallis.

Within each patient (controls, CRSsNP or CRSwNP), Th17 cells derived from tissue (Figures 4A–C) and from blood (Figures 4D–F) produced equal amounts of cytokines, including IL-17A, IL-17F, IL-21, and IL-22. Albeit not statistically significant, IL-21 was the most abundant cytokine produced by Th17 cells in the blood of all patient categories and in the tissue of control and CRSsNP patients whilst in the tissue of CRSwNP patients, IL-17A was the most abundant cytokine produced by Th17 cells.

### Th-17 cells produce less IL-21 in allergic CRSwNP

**Figure 5:**
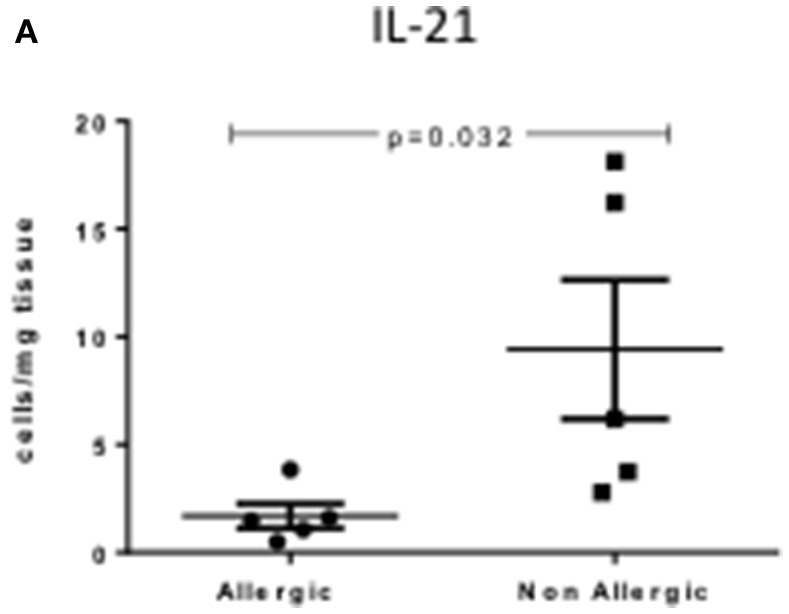
Percentage of IL-21+Th17 cells per mg of tissue in allergic and non-allergic CRSwNP mucosa. Medians with interquartile range. *P* ≤ 0.05 Kruskal-Wallis.

Allergic CRSwNP patients had decreased numbers of IL-21 producing Th17 cells compared to non-allergic CRSwNP (1.69 ± 0.57 vs. 9.41 ± 3.23) per mg of tissue respectively, Kruskal-Wallis *p* < 0.05 (Figure 5). No differences were seen in CRSsNP patients or in the peripheral blood of any of the patient groups.

## Discussion

This is the first study to our knowledge that characterizes the cytokine production by Th17 cells in CRS. It supports the findings of our previous study that demonstrated an increase in Th17 cells in CRSwNP patients compared to CRSsNP and controls (Miljkovic et al., 2016). By defining the specific cytokine production by Th17 cells we believe we now have increased understanding of the complex immune microenvironment characteristic of CRSwNP patients. CRS is divided into two disease phenotypes based on the presence or absence of nasal polyps. According to the current European position paper (EPOS) (Fokkens et al., 2012b) on CRS, nasal polyps should be visualized endoscopically, arising bilaterally from the middle meatus into the nasal cavity, allowing the classification of CRS patients into CRS with nasal polyps (CRSwNP) and CRS without nasal polyps (CRSsNP) (Fokkens et al., 2012b). Nasal polyps are grape-like structures which are composed of edema, inflammatory cells, connective tissue, mucous glands, and capillaries encased in pseudostratified epithelium (Bachert et al., 1997). In agreement with our study, in western countries, CRSwNP is thought to differ from CRSsNP not only macroscopically, but also by having a more eosinophilic Th2 type disease as opposed to Asian CRSwNP counterparts that have more of a neutrophilic inflammatory infiltrate (Van Zele et al., 2006; Zhang et al., 2008).

Th17 cells producing IL-17A were increased in CRSwNP patient polyps and peripheral blood compared to CRSsNP and controls. This IL-17A elevation in Th17 cells could account for the IL-17A abundance seen in CRSwNP patients in previously published studies (Makihara et al., 2010; Hu et al., 2013). In Japanese populations CRSwNP increases in IL-17A cytokine production are correlated with eosinophil numbers (Makihara et al., 2010; Saitoh et al., 2010). A different cohort of Chinese patients has linked IL-17A abundance with elevated amounts of neutrophils in CRSwNP (Hu et al., 2013). IL-17A is known to cause the induction and activation of neutrophils and neutrophil activating cytokines in the airways by inducing the release of chemokines that can recruit neutrophils and fungicidal peptides (Laan et al., 1999; Puel et al., 2012). Most recently, dysregulated IL-17A production was also found to promote neutrophil infiltration resulting in the delay of wound healing and tissue repair in mouse models, and could account for the inflammation and airway remodeling seen in certain CRSwNP patient subtypes (Takagi et al., 2016). IL-17F, although newly discovered and less studied, is very similar in structure to IL-17A and has a similar role (Chang et al., 2011). This is evident in the accumulation of neutrophils in allergic diseases with high IL-17A and IL-17F production (Kawaguchi et al., 2001). In our study, IL-17F, much like its IL-17A counterpart was increased in the polyps and peripheral blood of CRSwNP patients. Recently, however, *in vivo* studies of an induced model of colitis have shown that IL-17A and IL-17F can have differing roles. Mice deficient in IL-17F but not IL-17A have defective airway neutrophils in response to allergen challenge demonstrating an important functional difference in the IL-17F immune response which warrants further research (Yang et al., 2008).

Studies in CRS have reported increased levels of IL-21 mRNA and protein using ELISA in polyp tissues and peripheral blood. Our study adds to this research suggesting the increase may, in fact, be due to increases of IL-21 producing Th17 cells in CRSwNP patients (Xiao et al., 2015). IL-21 regulates T and B lymphocyte survival, activation and proliferation and *in vitro* IL-21 has been shown to have an effect on polyp B cell differentiation and IgG and IgA production (Zotos et al., 2010; Sarra et al., 2011; Xiao et al., 2015). In our study, CRSwNP patients that were atopic had remarkably less IL-21 cytokine producing Th17 cells as evidenced by flow cytometry compared to non-atopic CRSwNP. These results are in agreement with studies in allergic rhinitis: IL-21 administered at the time of antigen challenge in ovalbumin-induced mice reduces allergic symptoms and antigen-specific IgE levels (Hiromura et al., 2007). Further to this, signaling through the IL-21 receptor mediates house dust mite airway hyper-responsiveness by enhancing Th2 cytokine production (Lajoie et al., 2014). There are discrepancies, however, with other in vivo animal studies suggesting that IL-21 doesn't affect airway remodeling (Chen et al., 2015). Further studies, focusing on airway remodeling in CRS will be needed to elucidate the role of the Th17 cytokine IL-21 in allergic patients.

Few studies have investigated the role of IL-22 in CRS. Ramanthan et al confirmed the presence of the IL-22 receptor IL-22R1 on the surface of nasal epithelial cells and discovered lower receptor quantities in recalcitrant CRSwNP compared to CRSsNP and controls (Ramanathan et al., 2007). This was confirmed by another study by Wang et al however, there are conflicting reports about cytokine IL-22 levels, with one study showing no statistical difference in IL-22 levels between patient groups and the other finding IL-22 was significantly higher in CRSsNP mucosa compared to controls (Ramanathan et al., 2007; Wang et al., 2014). IL-22 is produced by activated T cells as well as innate lymphoid cells and innate immune cells. In our study, we show that Th17 derived IL-22 is increased in CRSwNP patients polyps and peripheral blood (Rutz et al., 2013). The cytokine is a key mediator of mucosal host defense and protects against extracellular pathogens (Zheng et al., 2008). IL-22 promotes keratinocyte migration and innate immune function as well as tissue repair (Wolk et al., 2004). Interestingly although IL-22 plays a protective role in mucosal diseases such as inflammatory bowel disease by enhancing barrier integrity of the intestinal tract, in other diseases such as psoriasis it has been eluded to synergise with proinflammatory cytokines and induce disease progression (Li et al., 2014; Cochez et al., 2016). A limitation of this study is the low number of control patients due to the lower amount of patients undergoing endoscopic skull base procedures compared to CRS patients; that were also not on corticosteroids or had any endoscopic or radiologic evidence of past or present sinonasal disease. Future studies will aim to characterize the cause of Th17 cell abundance by stimulating sinonasal tissue with suspected antigens involved in the pathogenesis of CRS.

As in this study, increased numbers of activated Th17 cells have been found in severe chronic inflammatory diseases such as asthma (Wong et al., 2009) and rheumatoid arthritis (Chen et al., 2011). It is evident that cytokines may have opposing effects in diverse tissue microenvironments and further functional studies are needed in order to assess whether the Th-17 derived cytokines we see elevated in CRSwNP patients' tissue and blood are in fact playing a protective or pathogenic role in CRS.

## Conclusion

In summary, although Th17 cells were increased in CRSwNP polyps and not in the periphery, Th17 cells producing IL-17A, IL-17F, IL21, and IL22 were significantly increased in CRSwNP patient polyps and peripheral blood suggesting an altered activation status of those cells both locally and systemically in CRSwNP patients. Notably, atopic CRSwNP has decreased amounts of Th17 derived IL-21 in their polyps implying a potential protective role for IL-21 in CRSwNP allergic inflammation.

## Author contributions

DM, Experimental techniques, manuscript writing. AP, Experimental design. manuscript editing. PW, Experimental design, manuscript editing. SV, Experimental design, manuscript editing.

### Conflict of interest statement

The authors declare that the research was conducted in the absence of any commercial or financial relationships that could be construed as a potential conflict of interest.

## Supplementary material

Supplementary Figure S1 Gating strategy. CD45+ cell were identified **(A)** and CD4+CD45+ **(B)** cells gated to identify CCR6+ Th17 cells **(C)**. Th17 cells further gated to reveal IL-17A **(D)**, IL-17F **(E)**, IL-21 **(F)**, and IL-22 **(G)** cytokines.

## References

[B1] Bachert C. Wagenmann M. Hauser U. Rudack C. (1997). IL-5 synthesis is upregulated in human nasal polyp tissue. J. Allergy Clin. Immunol. 99, 837–842. 10.1016/S0091-6749(97)80019-X 9215253

[B2] Brucklacher-Waldert V. Steinbach K. Lioznov M. Kolster M. Hölscher C. Tolosa E. (2009). Phenotypical characterization of human Th17 cells unambiguously identified by surface IL-17A expression. J. Immunol. 183, 5494–5501. 10.4049/jimmunol.0901000 19843935

[B3] Cao P. P. Li H. B. Wang B. F. Wang S. B. You X. J. Cui Y. H.. (2009). Distinct immunopathologic characteristics of various types of chronic rhinosinusitis in adult Chinese. J. Allergy Clin. Immunol. 124, 478–484, 484 e471–472. 10.1016/j.jaci.2009.05.017 19541359

[B4] Chang S. H. Dong C. Chang S. H. Dong C. (2011). Signaling of interleukin-17 family cytokines in immunity and inflammation. Cell. Signal. 23, 1069–1075. 10.1016/j.cellsig.2010.11.022 21130872 PMC3078175

[B5] Chen D. Y. Chen Y. M. Chen H. H. Hsieh C. W. Lin C. C. Lan J. L. (2011). Increasing levels of circulating Th17 cells and interleukin-17 in rheumatoid arthritis patients with an inadequate response to anti-TNF-alpha therapy. Arthritis Res. Ther. 13, R126. 10.1186/ar3431 21801431 PMC3239366

[B6] Chen H. Cheng S. Wang A. Bunjhoo H. Cao Y. Xie J.. (2015). IL-21 does not involve in OVA-induced airway remodeling and chronic airway inflammation. Int. J. Clin. Exp. Med. 8, 10640–10645. 26379855 PMC4565238

[B7] Cochez P. M. Michiels C. Hendrickx E. Van Belle A. B. Lemaire M. M. Dauguet N.. (2016). AhR modulates the IL-22-producing cell proliferation/recruitment in imiquimod-induced psoriasis mouse model. Eur. J. Immunol. 46, 1449–1459. 10.1002/eji.201546070 27000947

[B8] Coffelt S. B. Kersten K. Doornebal C. W. Weiden J. Vrijland K. Hau C. S.. (2015). IL-17-producing gammadelta T cells and neutrophils conspire to promote breast cancer metastasis. Nature 522, 345–348. 10.1038/nature14282 25822788 PMC4475637

[B9] De Nitto D. Sarra M. Pallone F. Monteleone G. (2010). Interleukin-21 triggers effector cell responses in the gut. World J. Gastroenterol. 16, 3638–3641. 10.3748/wjg.v16.i29.3638 20677335 PMC2915423

[B10] Fokkens W. J. Lund V. J. Mullol J. Bachert C. Alobid I. Baroody F. (2012a). European position paper on rhinosinusitis and nasal polyps. Rhinol. Suppl. 3, 1–298. 22764607

[B11] Fokkens W. J. Lund V. J. Mullol J. Bachert C. Alobid I. Baroody F.. (2012b). EPOS: European position paper on rhinosinusitis and nasal polyps 2012. a summary for otorhinolaryngologists. Rhinology 50, 1–12. 10.4193/Rhino50E2 22469599

[B12] Fossiez F. Djossou O. Chomarat P. Flores-Romo L. Ait-Yahia S. Maat C.. (1996). T cell interleukin-17 induces stromal cells to produce proinflammatory and hematopoietic cytokines. J. Exp. Med. 183, 2593–2603. 10.1084/jem.183.6.2593 8676080 PMC2192621

[B13] Hiromura Y. Kishida T. Nakano H. Hama T. Imanishi J. Hisa Y.. (2007). IL-21 administration into the nostril alleviates murine allergic rhinitis. J. Immunol. 179, 7157–7165. 10.4049/jimmunol.179.10.7157 17982108

[B14] Hu X. D. Bao Y. Y. Zhou S. H. Yao H. T. Mao J. Y. Ji X. X.. (2013). Interleukin-17A expression in patients with chronic rhinosinusitis and its relationship with clinical features. J. Int. Med. Res. 41, 777–784. 10.1177/0300060513478089 23613503

[B15] Hueber W. Patel D. D. Dryja T. Wright A. M. Koroleva I. Bruin G.. (2010). Effects of AIN457, a fully human antibody to interleukin-17A, on psoriasis, rheumatoid arthritis, and uveitis. Sci. Transl. Med. 2, 52ra72. 10.1126/scitranslmed.3001107 20926833

[B16] Kawaguchi M. Onuchic L. F. Li X. D. Essayan D. M. Schroeder J. Xiao H. Q.. (2001). Identification of a novel cytokine, ML-1, and its expression in subjects with asthma. J. Immunol. 167, 4430–4435. 10.4049/jimmunol.167.8.4430 11591768

[B17] Kim K. Kim G. Kim J. Y. Yun H. J. Lim S. C. Choi H. S. (2014). Interleukin-22 promotes epithelial cell transformation and breast tumorigenesis via MAP3K8 activation. Carcinogenesis 35, 1352–1361. 10.1093/carcin/bgu044 24517997

[B18] Kinugasa T. Gu X. B. Yang H. Reinecker H. C. (2000). Extracellular regulated mitogen activated kinase activity is required for the regulation of the intestinal epithelial cell barrier function by IL-17. Gastroenterology 118, A549–A549. 10.1016/S0016-5085(00)70351-9

[B19] Laan M. Cui Z. H. Hoshino H. Lötvall J. Sjöstrand M. Gruenert D. C.. (1999). Neutrophil recruitment by human IL-17 via C-X-C chemokine release in the airways. J. Immunol. 162, 2347–2352. 9973514

[B20] Lajoie S. Lewkowich I. Herman N. S. Sproles A. Pesce J. T. Wynn T. A.. (2014). IL-21 receptor signalling partially mediates Th2-mediated allergic airway responses. Clin. Exp. Allergy 44, 976–985. 10.1111/cea.12341 24807637 PMC4083497

[B21] Lee J. S. Tato C. M. Joyce-Shaikh B. Gulen M. F. Cayatte C. Chen Y.. (2015). Interleukin-23-Independent IL-17 production regulates intestinal epithelial permeability. Immunity 43, 727–738. 10.1016/j.immuni.2015.09.003 26431948 PMC6044435

[B22] Li L. J. Gong C. Zhao M. H. Feng B. S. (2014). Role of interleukin-22 in inflammatory bowel disease. World J. Gastroenterol. 20, 18177–18188. 10.3748/wjg.v20.i48.18177 25561785 PMC4277955

[B23] Lin S. Yang X. Liang D. Zheng S. G. (2014). Treg cells: a potential regulator for IL-22 expression? Int. J. Clin. Exp. Pathol. 7, 474–480. 24551268 PMC3925892

[B24] Lin S. Y. Nnacheta L. C. (2015). American academy of otolaryngology-head and neck surgery foundation (AAO-HNSF) will publish its latest “Clinical practice guideline: allergic rhinitis (AR) in February, 2015.” Am. J. Rhinol. Allergy 29, 82. 25590326 10.2500/ajra.2015.29.4142

[B25] Makihara S. Okano M. Fujiwara T. Kariya S. Noda Y. Higaki T.. (2010). Regulation and characterization of IL-17A expression in patients with chronic rhinosinusitis and its relationship with eosinophilic inflammation. J. Allergy Clin. Immunol. 126, 397–400, 400 e391–e311. 10.1016/j.jaci.2010.05.014 20621345

[B26] Miljkovic D. Bassiouni A. Cooksley C. Ou J. Hauben E. Wormald P. J.. (2014). Association between group 2 innate lymphoid cells enrichment, nasal polyps and allergy in chronic rhinosinusitis. Allergy 69, 1154–1161. 10.1111/all.12440 24924975

[B27] Miljkovic D. Psaltis A. Wormald P. J. Vreugde S. (2016). T regulatory and Th17 cells in chronic rhinosinusitis with polyps. Int. Forum Allergy Rhinol. 6, 82634. 10.1002/alr.21742 27012842

[B28] Nakou M. Papadimitraki E. D. Fanouriakis A. Bertsias G. K. Choulaki C. Goulidaki N.. (2013). Interleukin-21 is increased in active systemic lupus erythematosus patients and contributes to the generation of plasma B cells. Clin. Exp. Rheumatol. 31, 172–179. 23137515

[B29] Neurath M. F. (2014). Cytokines in inflammatory bowel disease. Nat. Rev. Immunol. 14, 329–342. 10.1038/nri3661 24751956

[B30] O'Connor W. Kamanaka M. Booth C. J. Town T. Nakae S. Iwakura Y.. (2009). A protective function for interleukin 17A in T cell-mediated intestinal inflammation. Nat. Immunol. 10, 603–609. 10.1038/ni.1736 19448631 PMC2709990

[B31] Pandya J. M. Lundell A. C. Hallström M. Andersson K. Nordström I. Rudin A. (2016). Circulating T helper and T regulatory subsets in untreated early rheumatoid arthritis and healthy control subjects. J. Leukoc. Biol. 100, 823–833. 10.1189/jlb.5A0116-025R 27190305

[B32] Puel A. Cypowyj S. Maródi L. Abel L. Picard C. Casanova J. L. (2012). Inborn errors of human IL-17 immunity underlie chronic mucocutaneous candidiasis. Curr. Opin. Allergy Clin. Immunol. 12, 616–622. 10.1097/ACI.0b013e328358cc0b 23026768 PMC3538358

[B33] Ramanathan M. Spannhake E. W. Lane A. P. (2007). Chronic rhinosinusitis with nasal polyps is associated with decreased expression of mucosal interleukin 22 receptor. Laryngoscope 117, 1839–1843. 10.1097/MLG.0b013e31811edd4f 17906500

[B34] Rutz S. Eidenschenk C. Ouyang W. (2013). IL-22, not simply a Th17 cytokine. Immunol. Rev. 252, 116–132. 10.1111/imr.12027 23405899

[B35] Saitoh T. Kusunoki T. Yao T. Kawano K. Kojima Y. Miyahara K.. (2010). Role of interleukin-17A in the eosinophil accumulation and mucosal remodeling in chronic rhinosinusitis with nasal polyps associated with asthma. Int. Arch. Allergy Immunol. 151, 8–16. 10.1159/000232566 19672092

[B36] Sarra M. Franzè E. Pallone F. Monteleone G. (2011). Targeting interleukin-21 in inflammatory diseases. Expert Opin. Ther. Targets 15, 695–702. 10.1517/14728222.2011.561319 21391901

[B37] Siloşi I. Boldeanu M. V. Cojocaru M. Biciuşcă V. Pădureanu V. Bogdan M.. (2016). The relationship of cytokines IL-13 and IL-17 with autoantibodies profile in early rheumatoid arthritis. J. Immunol. Res. 2016:3109135. 10.1155/2016/3109135 27579330 PMC4989068

[B38] Takagi N. Kawakami K. Kanno E. Tanno H. Takeda A. Ishii K.. (2016). IL-17A promotes neutrophilic inflammation and disturbs acute wound healing in skin. Exp. Dermatol. 26, 137–144. 10.1111/exd.13115 27305096

[B39] Van Bruaene N. Pérez-Novo C. A. Basinski T. M. Van Zele T. Holtappels G. De Ruyck N.. (2008). T-cell regulation in chronic paranasal sinus disease. J. Allergy Clin. Immunol. 121, 1435–1441, 1441 e1431–1433. 10.1016/j.jaci.2008.02.018 18423831

[B40] Van Zele T. Claeys S. Gevaert P. Van Maele G. Holtappels G. Van Cauwenberge P.. (2006). Differentiation of chronic sinus diseases by measurement of inflammatory mediators. Allergy 61, 1280–1289. 10.1111/j.1398-9995.2006.01225.x 17002703

[B41] Wang X. Gao M. Xu Y. Guo H. Zhao C. (2014). Expression of interleukin-22 and its significance in the pathogenesis of chronic rhinosinusitis. Int. J. Clin. Exp. Pathol. 7, 5709–5716. 25337212 PMC4203183

[B42] Wei P. Hu G. H. Kang H. Y. Yao H. B. Kou W. Zhang C.. (2014). Role of the Aryl hydrocarbon receptor in the pathogenesis of chronic rhinosinusitis with nasal polyps. Inflammation 37, 387–395. 10.1007/s10753-013-9751-7 24092408

[B43] Wolk K. Kunz S. Witte E. Friedrich M. Asadullah K. Sabat R. (2004). IL-22 increases the innate immunity of tissues. Immunity 21, 241–254. 10.1016/j.immuni.2004.07.007 15308104

[B44] Wong C. K. Lun S. W. Ko F. W. Wong P. T. Hu S. Q. Chan I. H.. (2009). Activation of peripheral Th17 lymphocytes in patients with asthma. Immunol. Invest. 38, 652–664. 10.1080/08820130903062756 19811428

[B45] Xiao L. Wei Y. Zhang Y. N. Luo X. Yang B. Y. Yu S. F.. (2015). Increased IL-21 expression in chronic rhinosinusitis with nasalpolyps. Clin. Exp. Allergy 45, 404–413. 10.1111/cea.12475 25495679

[B46] Yang X. O. Chang S. H. Park H. Nurieva R. Shah B. Acero L.. (2008). Regulation of inflammatory responses by IL-17F. J. Exp. Med. 205, 1063–1075. 10.1084/jem.20071978 18411338 PMC2373839

[B47] Yu J. He S. Liu P. Hu Y. Wang L. Wang X.. (2015). Interleukin21 promotes the development of ulcerative colitis and regulates the proliferation and secretion of follicular T helper cells in the colitides microenvironment. Mol. Med. Rep. 11, 1049–1056. 10.3892/mmr.2014.2824 25371082

[B48] Zhang N. Van Zele T. Perez-Novo C. Van Bruaene N. Holtappels G. DeRuyck N.. (2008). Different types of T-effector cells orchestrate mucosal inflammation in chronic sinus disease. J. Allergy Clin. Immunol. 122, 961–968. 10.1016/j.jaci.2008.07.008 18804271

[B49] Zheng Y. Valdez P. A. Danilenko D. M. Hu Y. Sa S. M. Gong Q.. (2008). Interleukin-22 mediates early host defense against attaching and effacing bacterial pathogens. Nat. Med. 14, 282–289. 10.1038/nm1720 18264109

[B50] Zotos D. Coquet J. M. Zhang Y. Light A. D'Costa K. Kallies A.. (2010). IL-21 regulates germinal center B cell differentiation and proliferation through a B cell-intrinsic mechanism. J. Exp. Med. 207, 365–378. 10.1084/jem.20091777 20142430 PMC2822601

