# Supplementary figures and images for: Chronic Rhinosinusitis with Polyps Is Characterized by Increased Mucosal and Blood Th17 Effector Cytokine Producing Cells
Source: Front Physiol. 2017 Dec 19;8:898. doi: 10.3389/fphys.2017.00898 (PMC5742278; doi:10.3389/fphys.2017.00898)

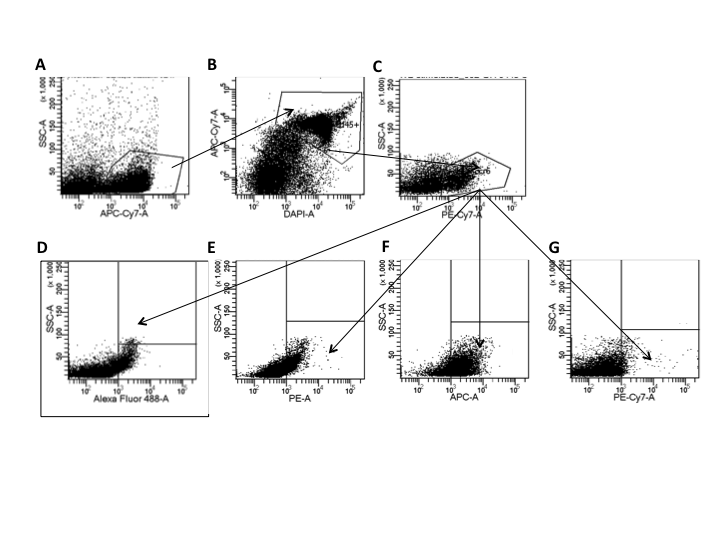

Supplement: Supplementary Figure S1 — Gating strategy. CD45+ cell were identified (A) and CD4+CD45+ (B) cells gated to identify CCR6+ Th17 cells (C). Th17 cells further gated to reveal IL-17A (D), IL-17F (E), IL-21 (F), and IL-22 (G) cytokines. [file Image1.tiff]
