# Supplementary material for: Chronic Rhinosinusitis with Polyps Is Characterized by Increased Mucosal and Blood Th17 Effector Cytokine Producing Cells
Source: Front Physiol. 2017 Dec 19;8:898. doi: 10.3389/fphys.2017.00898 (PMC5742278; doi:10.3389/fphys.2017.00898)
Supplement: Supplementary file 2 [file Table1.docx]

Supplementary Table S1. Flow cytometry antibodies

| Target Antigen | Clone | Conjugation |
| --- | --- | --- |
| CD45 | H130 | APC-eFluor-780 |
| CD4 | RPA-T4 | Ef-450 |
| CCR6 | R6H1 | Pe-Cy7 |
| IL-17A | EBio64DEC17 | FITC |
| IL-17F | SHCR17 | PE |
| IL-21 | EBio3A3-N2 | eFluor-660 |
| IL-22 | 22URTI | Percp-eFluor-710 |
