# Supplementary material for: Chronic Rhinosinusitis with Polyps Is Characterized by Increased Mucosal and Blood Th17 Effector Cytokine Producing Cells
Source: Front Physiol. 2017 Dec 19;8:898. doi: 10.3389/fphys.2017.00898 (PMC5742278; doi:10.3389/fphys.2017.00898)
Supplement: Supplementary file 3 [file Table2.docx]

Supplementary Table S2. Patient demographics

|  | Controls | CRSsNP | CRSwNP |
| --- | --- | --- | --- |
| Number | 5 | 19 | 12 |
| Median Age (IQR) | 54 (52-55) | 59 (50-65) | 56 (51-61) |
| Male/Female | 2/3 | 13/6 | 10/2 |
| Asthmatic/non-asthmatic | 0/5 | 6/13 | 6/6 |
| Allergic/non-allergic | 0/5 | 5/14 | 5/7 |
| Previous ESS: 0/1/≥ 2 | N/A | 10/6/3 | 6//2/4 |
