# Supplementary material for: Chronic Rhinosinusitis with Polyps Is Characterized by Increased Mucosal and Blood Th17 Effector Cytokine Producing Cells
Source: Front Physiol. 2017 Dec 19;8:898. doi: 10.3389/fphys.2017.00898 (PMC5742278; doi:10.3389/fphys.2017.00898)
Supplement: Supplementary file 4 [file Table3.docx]

Supplementary Table S3. Flow cytometry analysis of IL-17A, IL17-F, IL-21 and IL-22 expressing Th17 cells in tissue (Kruskal-Wallis mean +/- SEM)

|  | Controls | CRSsNP | CRSwNP |
| --- | --- | --- | --- |
| Th17+IL-17A (per mg of tissue) | 0.77 +/- 0.07 | 0.11 +/ -0.43 | 9.53 +/- 2.71 |
| Th17+IL-17F (per mg of tissue) | 0.56 +/- 0.04 | 0.88 +/-0.31 | 4.96 +/- 1.48 |
| Th17+IL-21 (per mg of tissue) | 1.53 +/- 0.55 | 1.60 +/- 0.71 | 5.55 +/- 2.01 |
| Th17+IL-22 (per mg of tissue) | 0.88 +/- 0.26 | 0.70+/-0.28 | 4.73 +/- 1.58 |
