# Supplementary material for: Chronic Rhinosinusitis with Polyps Is Characterized by Increased Mucosal and Blood Th17 Effector Cytokine Producing Cells
Source: Front Physiol. 2017 Dec 19;8:898. doi: 10.3389/fphys.2017.00898 (PMC5742278; doi:10.3389/fphys.2017.00898)
Supplement: Supplementary file 5 [file Table4.docx]

Supplementary Table S4. Flow cytometry analysis of IL-17A, IL17-F, IL-21 and IL-22 expressing Th17 cells in peripheral blood (Kruskal-Wallis mean +/- SEM)

|  | Controls | CRSsNP | CRSwNP |
| --- | --- | --- | --- |
| Th17+IL-17A (% CD45) | 0.73 +/- 0.33 | 1.55+/-0.62 | 3.31 +/- 0.93 |
| Th17+IL-17F (% CD45) | 0.56 +/- 0.04 | 0.88 +/- 0.31 | 4.97 +/- 1.48 |
| Th17+IL-21 (% CD45) | 1.43 +/- 0.57 | 2.46 +/- 0.88 | 5.48 +/- 1.62 |
| Th17+IL-22 (% CD45) | 0.39 +/- 0.16 | 1.32 +/- 0.74 | 3.01 +/- 1.12 |
